# Supplementary material for: The effect of whole-body vibration on lower extremity function in children with cerebral palsy: A meta-analysis
Source: PLoS One. 2023 Mar 10;18(3):e0282604. doi: 10.1371/journal.pone.0282604 (PMC10004558; doi:10.1371/journal.pone.0282604)
Supplement: S1 Table — (DOCX) [file pone.0282604.s002.docx]

S1 Table. Search strategy.

**Pubmed:**

| **No.** | **Query** | **Results** |
| --- | --- | --- |
| **#5** | #1 AND #2 AND #3 AND #4 | 10 |
| **#4** | (((Lower Extremity[Mesh]) OR (Lower Limb*[All Fields])) OR (Membrum inferius[All Fields])) OR (Lower Extremit*[All Fields]) | 267,230 |
| **#3** | (Children[Mesh]) OR (Child[All Fields]) | 2,890,644 |
| **#2** | ((((((((((((((((((((CP (Cerebral Palsy[Mesh])) OR (CP[All Fields])) OR (Dystonic-Rigid Cerebral Pals*[All Fields])) OR (Mixed Cerebral Pals*[All Fields])) OR (Monoplegic Infantile Cerebral Pals*[All Fields])) OR (Quadriplegic Infantile Cerebral Pals*[All Fields])) OR (Rolandic Type Cerebral Pals*[All Fields])) OR (Congenital Cerebral Pals*[All Fields])) OR (Little Disease[All Fields])) OR (Little's Disease[All Fields])) OR (Spastic Diplegia[All Fields])) OR (Monoplegic Cerebral Pals*[All Fields])) OR (Athetoid Cerebral Pals*[All Fields])) OR (Dyskinetic Cerebral Pals*[All Fields])) OR (Atonic Cerebral Pals*[All Fields])) OR (Hypotonic Cerebral Pals*[All Fields])) OR (Diplegic Infantile Cerebral Palsy[All Fields])) OR (Spastic Cerebral Pals*[All Fields])) | 152,055 |
| **#1** | (whole body vibration[Mesh]) OR WBV[All Fields]) | 2,866 |

**Web of science:**

| **No.** | **Query** | **Results** |
| --- | --- | --- |
| **#5** | #1 AND #2 AND #3 AND #4 | 16 |
| **#4** | ALL=((Lower Extremit*) OR (Lower Limb*)) OR (Membrum inferius)) | 231,278 |
| **#3** | ALL=(Child*) | 3,516,917 |
| **#2** | ALL=((((((((((((((((((((((CP (Cerebral Palsy)) OR (Dystonic-Rigid Cerebral Pals*)) OR (Mixed Cerebral Pals*)) OR (Monoplegic Infantile Cerebral Palsy)) OR (Quadriplegic Infantile Cerebral Palsy)) OR (Rolandic Type Cerebral Palsy)) OR (Congenital Cerebral Palsy)) OR (Little Disease)) OR (Little's Disease)) OR (Spastic Diplegia)) OR (Monoplegic Cerebral Palsy)) OR (Athetoid Cerebral Palsy)) OR (Dyskinetic Cerebral Palsy)) OR (Atonic Cerebral Palsy)) OR (Hypotonic Cerebral Pals*)) OR (Diplegic Infantile Cerebral Palsy)) OR (Spastic Cerebral Pals*)) OR (Monoplegic Cerebral Pals*)) OR (Cerebral Palsy)) | 1,743,383 |
| **#1** | ALL=(whole body vibration) OR (WBV) | 6,469 |

**EBSCO:**

| **No.** | **Query** | **Results** |
| --- | --- | --- |
| **#1** | TX (whole body vibration) OR (WBV) AND TX ((((((((((((((((((CP (Cerebral Palsy)) OR (Dystonic-Rigid Cerebral Pals*)) OR (Mixed Cerebral Pals*)) OR (Monoplegic Infantile Cerebral Palsy)) OR (Quadriplegic Infantile Cerebral Palsy)) OR (Rolandic Type Cerebral Palsy)) OR (Congenital Cerebral Palsy)) OR (Little Disease)) OR (Little's Disease)) OR (Spastic Diplegia)) OR (Monoplegic Cerebral Palsy)) OR (Athetoid Cerebral Palsy)) OR (Dyskinetic Cerebral Palsy)) OR (Atonic Cerebral Palsy)) OR (Hypotonic Cerebral Pals*)) OR (Diplegic Infantile Cerebral Palsy)) OR (Spastic Cerebral Pals*)) OR (Monoplegic Cerebral Pals*)) OR (Cerebral Palsy)) AND TX (Child*) AND TX ((Lower Extremit*) OR (Lower Limb*)) OR (Membrum inferius) | 161 |

**Cochrane:**

| **No.** | **Query** | **Results** |
| --- | --- | --- |
| **#1** | All Text (whole body vibration) OR (WBV) AND All Text ((((((((((((((((((CP (Cerebral Palsy)) OR (Dystonic-Rigid Cerebral Pals*)) OR (Mixed Cerebral Pals*)) OR (Monoplegic Infantile Cerebral Palsy)) OR (Quadriplegic Infantile Cerebral Palsy)) OR (Rolandic Type Cerebral Palsy)) OR (Congenital Cerebral Palsy)) OR (Little Disease)) OR (Little's Disease)) OR (Spastic Diplegia)) OR (Monoplegic Cerebral Palsy)) OR (Athetoid Cerebral Palsy)) OR (Dyskinetic Cerebral Palsy)) OR (Atonic Cerebral Palsy)) OR (Hypotonic Cerebral Pals*)) OR (Diplegic Infantile Cerebral Palsy)) OR (Spastic Cerebral Pals*)) OR (Monoplegic Cerebral Pals*)) OR (Cerebral Palsy)) AND All Text (Child*) AND All Text ((Lower Extremit*) OR (Lower Limb*)) OR (Membrum inferius) | 20 |

**EMBASE:**

| **No.** | **Query** | **Results** |
| --- | --- | --- |
| **#5** | #1 AND #2 AND #3 AND #4 | 10 |
| **#4** | All Fields='Lower Extremit*' OR 'Lower Limb*' OR 'Membrum inferius' | 198,443 |
| **#3** | All Fields='Child*' | 3,629,200 |
| **#2** | All Fields= 'Cerebral Palsy' | 47,915 |
| **#1** | All Fields='whole body vibration' OR 'WBV' | 3,927 |

**Scopus:**

| **No.** | **Query** | **Results** |
| --- | --- | --- |
| **#5** | #1 AND #2 AND #3 AND #4 | 238 |
| **#4** | ALL((Lower Extremit*) OR (Lower Limb*)) OR (Membrum inferius) | 489,365 |
| **#3** | ALL(Child*) | 7,094,999 |
| **#2** | ALL(((((((((((((((((((CP (Cerebral Palsy)) OR (Dystonic-Rigid Cerebral Pals*)) OR (Mixed Cerebral Pals*)) OR (Monoplegic Infantile Cerebral Palsy)) OR (Quadriplegic Infantile Cerebral Palsy)) OR (Rolandic Type Cerebral Palsy)) OR (Congenital Cerebral Palsy)) OR (Little Disease)) OR (Little's Disease)) OR (Spastic Diplegia)) OR (Monoplegic Cerebral Palsy)) OR (Athetoid Cerebral Palsy)) OR (Dyskinetic Cerebral Palsy)) OR (Atonic Cerebral Palsy)) OR (Hypotonic Cerebral Pals*)) OR (Diplegic Infantile Cerebral Palsy)) OR (Spastic Cerebral Pals*)) OR (Monoplegic Cerebral Pals*)) OR (Cerebral Palsy)) | 2,955,096 |
| **#1** | ALL(whole body vibration) OR (WBV) | 25,308 |

**Wanfang：**

| **No.** | **Query** | **Results** |
| --- | --- | --- |
| **#1** | All fields=(whole body vibration) AND All fields= (children with cerebral palsy) AND All fields= (lower extremity function) | 6 |

**CNKI：**

| **No.** | **Query** | **Results** |
| --- | --- | --- |
| **#1** | Theme=(whole body vibration) AND Theme=(children with cerebral palsy) AND Theme=(lower extremity function) | 7 |

**VIP:**

| **No.** | **Query** | **Results** |
| --- | --- | --- |
| **#1** | All fields=(whole body vibration) AND All fields=(children with cerebral palsy) AND All fields=(lower extremity function) | 4 |
