# Supplementary figures and images for: The effect of whole-body vibration on lower extremity function in children with cerebral palsy: A meta-analysis
Source: PLoS One. 2023 Mar 10;18(3):e0282604. doi: 10.1371/journal.pone.0282604 (PMC10004558; doi:10.1371/journal.pone.0282604)

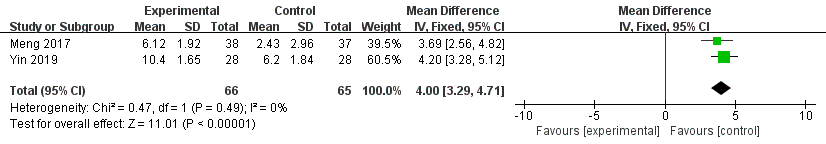

Supplement: S1 Fig — (TIF) [file pone.0282604.s003.tif]

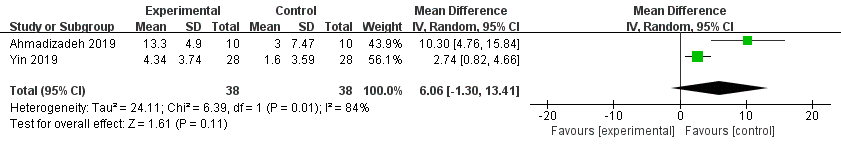

Supplement: S2 Fig — (TIF) [file pone.0282604.s004.tif]

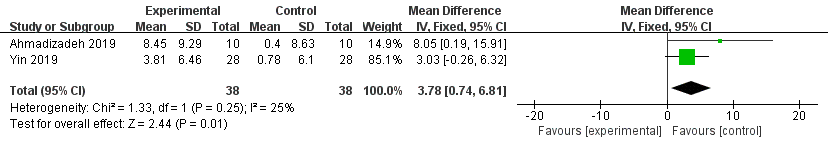

Supplement: S3 Fig — (TIF) [file pone.0282604.s005.tif]

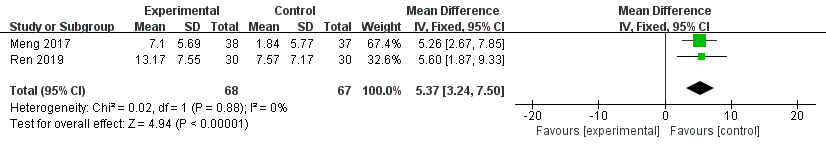

Supplement: S4 Fig — (TIF) [file pone.0282604.s006.tif]

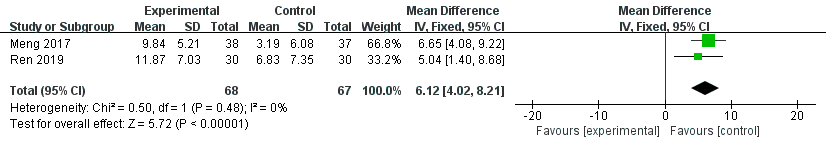

Supplement: S5 Fig — (TIF) [file pone.0282604.s007.tif]
